# Nonuremic penile calciphylaxis treated with pentoxifylline

**DOI:** 10.1016/j.jdcr.2025.06.044

**Published:** 2025-07-19

**Authors:** Riddhi D. Patel, Simran Chadha, Karishma Daftary, Lida Zheng

**Affiliations:** a Northwestern University Feinberg School of Medicine, Chicago, Illinois; b Department of Dermatology, Northwestern University Feinberg School of Medicine, Chicago, Illinois

**Keywords:** calciphylaxis, nonuremic, penile, pentoxifylline

## Introduction

Calciphylaxis is characterized by cutaneous arteriolar calcification and is classically associated with end-stage renal disease, although it may occur in patients with normal renal function or earlier stages of chronic kidney disease. Here, we report a case of nonuremic penile calciphylaxis with excellent response to pentoxifylline.

## Case report

A 74-year-old man with a history of heart failure, coronary artery disease, and diabetes mellitus presented to the emergency room with nonhealing penile ulcers. The patient reported the ulcers first developed 7 months prior and gradually enlarged. The lesions caused severe pain and he experienced alteration in his urinary stream, raising concern for urethral involvement. He had been previously treated with topical and oral steroids for a presumed diagnosis of erosive genital lichen planus, but did not note improvement with these therapies. He denied similar lesions elsewhere on the body and did not endorse fevers or chills. Notably, the patient did not have a history of chronic kidney disease or any exposure to warfarin.

**Fig 1:**
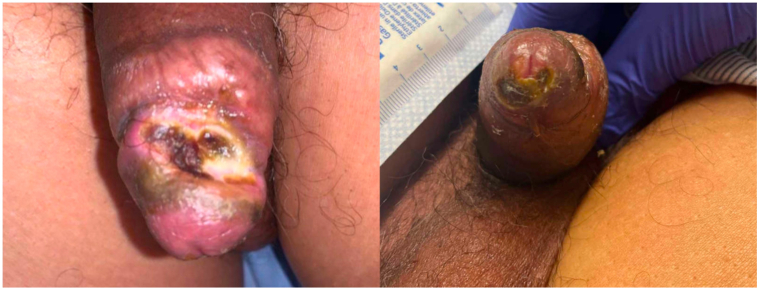
Examination demonstrated a deep ulceration of the dorsal glans penis with a fibrinous base and a shallower ulceration of the inferior urethral meatus.

Physical examination revealed a deep ulceration of the dorsal penile corona and distal shaft with rounded borders and a yellow, fibrinous base. There was a similar ulceration located on the ventral glans penis and urethral meatus (Fig 1). The patient did not have inguinal lymphadenopathy. Blood counts, kidney, and liver function were within normal limits. A superficial wound culture grew few group B streptococci. Computed tomography imaging demonstrated ulcerations of the penile tip measuring up to 1.3 centimeters and linear skin calcifications inferior to the lesions.

**Fig 2:**
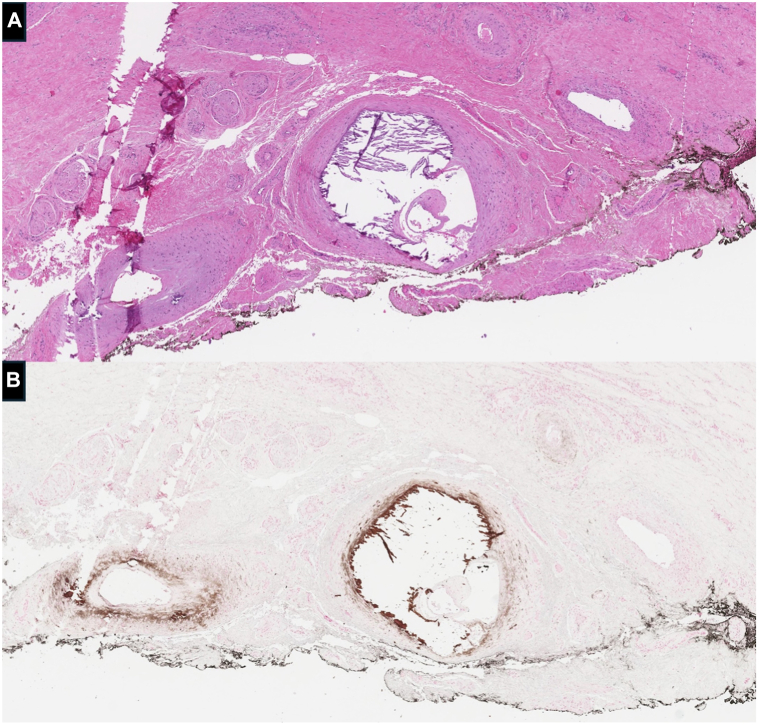
Biopsy of the ventral penile ulcer revealed **(A)** concentric calcifications of a deep dermal vessel, and **(B)** calcifications were further highlighted by Von Kossa staining (hematoxylin and eosin, 40× magnification).

Histopathologic evaluation revealed inflammatory fibronecrotic crust overlying an ulcer with marked calcification of arterioles and focal organized thrombi within the dermis. Von Kossa stain highlighted concentric vascular calcification of small-sized, medium-sized, and large-sized vessels (Fig 2, *A* and *B*). Thus, a diagnosis of nonuremic penile calciphylaxis was made.

**Fig 3:**
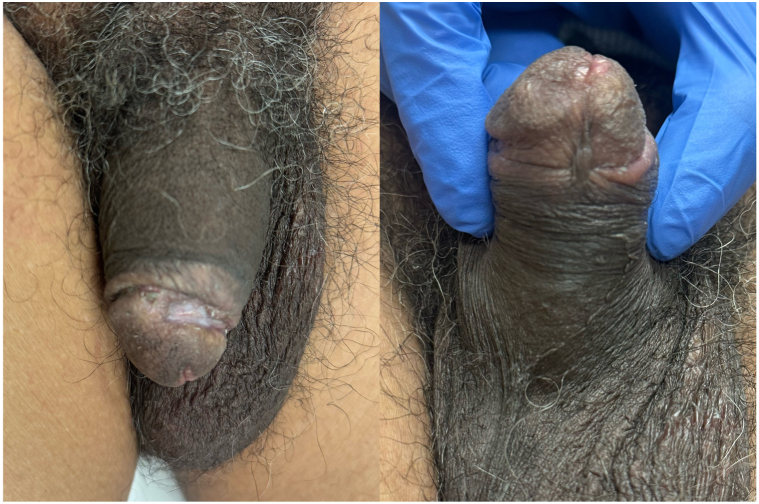
Examination revealed interval improvement in the size and depth of the ulceration of the dorsal glans penis and resolution of the ulceration of the inferior urethral meatus.

The patient was on dual antiplatelet therapy for his comorbid diagnoses. In addition, the patient was started on pentoxifylline 400 mg 3 times a day and diligent wound care with dilute vinegar soaks and topical antibiotics. Although we recommended starting Vitamin K 10 mg 3 times per week, the patient did not initiate this therapy. Intralesional sodium thiosulfate was considered but held in reserve due to the pain of injections. Fortunately, at the 1-month follow-up, the ulcer of the ventral glans penis had completely resolved, and the ulcer of the dorsal penis had significantly improved (Fig 3). The patient’s pain was considerably alleviated. Given excellent clinical response, the patient was continued on this regimen of pentoxifylline 400 mg 3 times per day. At the 6-month follow-up, the patient continued to report improvement in the appearance and size of the lesions. He noted that his pain had resolved with continued use of pentoxifylline and vinegar soaks 2 to 3 times weekly.

## Discussion

Calciphylaxis is classically associated with end-stage renal disease, particularly in individuals on hemodialysis due to dysregulation of calcium and phosphate metabolism. However, calciphylaxis can occur in patients with no renal impairment or with normal calcium and phosphate levels. Although the pathogenesis of calciphylaxis remains unclear, classic risk factors include diabetes, hypervitaminosis D, female sex, obesity, warfarin use, and protein C or S deficiency.^1^^,^^2^ Cases of nonuremic calciphylaxis have been documented with the most common causes being primary hyperparathyroidism, connective tissue diseases, alcoholic liver diseases, and malignancy, none of which were present in our patient.^3^ Our patient maintained calcium, phosphorus, and albumin levels within the normal range throughout his treatment course. In this case, the patient’s diabetes mellitus and coronary artery disease may have predisposed him to microvascular calcification despite the absence of other typical risk factors.

Treatment for calciphylaxis is multimodal, including options such as tissue plasminogen activator, sodium thiosulfate, bisphosphonates, and surgical interventions including debridement and parathyroidectomy.^4^ Avoidance of corticosteroids and warfarin are also necessary as both medications promote vascular calcification. Warfarin promotes vascular calcification by inhibiting Vitamin K which is needed to activate Matrix GIa proteins which actively prevent calcification. For this reason, vitamin K supplementation has been used to treat calciphylaxis in case reports. Penile calciphylaxis is particularly resistant to standard treatments.^5^ Intravenous or intralesional sodium thiosulfate has been used as the first-line therapy for penile calciphylaxis in multiple case reports due to its vasodilatory and antioxidant effects.^6^, ^7^, ^8^ Pain associated with intralesional sodium thiosulfate treatment can be addressed by mixing 1:1 with lidocaine. However, intravenous sodium thiosulfate was not preferred in this patient due to a baseline prolonged QT interval. Surgical options, including limited debridement, remain an option. Surgical treatment approaches, although effective, can come with significant psychosexual burden for patients who require partial or total penectomy.^7^

Pentoxifylline, a phosphodiesterase inhibitor, promotes vasodilation in the skeletal muscle vascular beds and inhibits leukocyte-derived free radicals, limiting ischemia-related tissue damage. Its use in peripheral vascular disease has been well documented, and few case reports describe successful treatment of calciphylaxis with pentoxifylline, resulting in complete remission of skin lesions.^9^^,^^10^ No reports exist documenting this as a treatment option for penile calciphylaxis; thus, this case adds to this body of literature for patients with penile involvement.

Prognosis and outcomes for patients with calciphylaxis remain poor. In patients without significant chronic kidney disease, 6-month survival is 72% with septicemia from infected wounds as the leading cause of death.^4^ Penile calciphylaxis has a mortality rate of 69% within 6 months.^7^ These statistics underscore the importance of wound care and prompt treatment of skin lesions to reduce mortality. Beyond survival, calciphylaxis causes significant morbidity, including severe pain, chronic wounds, and recurrent hospitalizations. Our patient experienced ongoing pain, despite improvement in skin lesions, and had recurrent hospitalizations before initiating pentoxifylline.

In conclusion, pentoxifylline may be a viable treatment option for penile nonuremic calciphylaxis, particularly in patients who have failed prior treatments or are unable to safely use intralesional or intravenous sodium thiosulfate. While this case highlights the potential efficacy of pentoxifylline, larger studies are needed to validate its effectiveness and establish its role in the treatment of calciphylaxis.

## Conflicts of interest

None disclosed.

## References

[1] Weenig R.H., Sewell L.D., Davis M.D., McCarthy J.T., Pittelkow M.R. (2007). Calciphylaxis: natural history, risk factor analysis, and outcome. J Am Acad Dermatol.

[2] Nigwekar S.U., Kroshinsky D., Nazarian R.M. (2015). Calciphylaxis: risk factors, diagnosis, and treatment. Am J Kidney Dis.

[3] Nigwekar S.U., Wolf M., Sterns R.H., Hix J.K. (2008). Calciphylaxis from nonuremic causes: a systematic review. Clin J Am Soc Nephrol.

[4] McCarthy J.T., El-Azhary R.A., Patzelt M.T. (2016). Survival, risk factors, and effect of treatment in 101 patients with calciphylaxis. Mayo Clin Proc.

[5] Wajih Z., Singer R. (2022). Successful treatment of calciphylaxis with vitamin K in a patient on haemodialysis. Clin Kidney J.

[6] Smilnak G., Jiang M., Jain B. (2022). Calciphylaxis of the penis and distal digits: a case report. J Med Case Rep.

[7] O'Neil B., Southwick A.W. (2012). Three cases of penile calciphylaxis: diagnosis, treatment strategies, and the role of sodium thiosulfate. Urology.

[8] Sandhu G., Gini M.B., Ranade A., Djebali D., Smith S. (2012). Penile calciphylaxis: a life-threatening condition successfully treated with sodium thiosulfate. Am J Ther.

[9] Tittelbach J., Graefe T., Wollina U. (2001). Painful ulcers in calciphylaxis - combined treatment with maggot therapy and oral pentoxyfillin. J Dermatolog Treat.

[10] Rrapi R., Chand S., Gabel C. (2021). Early diagnosis and intervention of calciphylaxis leading to rapid resolution. JAAD Case Rep.

